# Effects of Self-Weighing During Weight Loss Treatment: A 6-Month Randomized Controlled Trial

**DOI:** 10.3389/fpsyg.2020.00397

**Published:** 2020-03-10

**Authors:** Alberto Hernández-Reyes, Fernando Cámara-Martos, Ángela Vidal, Rafael Molina-Luque, Rafael Moreno-Rojas

**Affiliations:** ^1^Department of Bromatology and Food Technology, University of Córdoba, Córdoba, Spain; ^2^Department of Animal Medicine and Surgery, University of Córdoba, Córdoba, Spain; ^3^Department of Nursing, University of Córdoba, Córdoba, Spain

**Keywords:** obesity, weight-loss, self-weighing, goals, overweight

## Abstract

**Objective:**

To examine the effectiveness of self-weighing for weight loss in men for 6 months.

**Methods:**

In the present study, 54 men, mean age of 40.1 ± 11.1 years, with overweight or obesity, were recruited and randomly assigned into two groups: control group (CG), without weight self-monitoring and intervention group (IG), with weight self-monitoring. Both groups received the same nutritional and educational advice and the establishment of a weight target to reach in the weight loss program. Subjects of IG also had individualized motivating content to improve self-management for 24 weeks. Anthropometric indices were measured at baseline and weekly for 24 weeks.

**Results:**

When the group assigned after randomization was introduced in the analysis, its influence was significant in weight loss (*F*1.52 = 19.465, ± 2 = 0.272, *p* < 0.001) and in the decrease in body fat percentage (*F*1.52 = 8,306, ± 2 = 0.132, *p* < 0.01).

**Conclusion:**

Study results indicate that self-weighing can help patients to lose additional weight. Our findings have implications in the emerging area of the behavioral approach of patients undergoing weight-loss treatment, as well as clinical care processes.

**Clinical Trial Registration:**

www.ClinicalTrials.gov, identifier NCT04032249.

## Introduction

Evidence from randomized controlled clinical trials shows that weight loss programs that include behavioral therapies may involve significant weight loss in overweight or obese adults. These behavioral variables consist in self-monitoring dietary interventions (Turner-McGrievy et al., 2019), physical activity (Conroy et al., 2011; Van Hoye et al., 2015), and other cognitive aspects such as stimulus control or setting specific goals (Leung et al., 2019). Despite the potential of this tool, self-monitoring by the patient declines over time, failing to record the diet being carried out (Burke et al., 2011) or monitoring of physical activity. As previously reported by Campbell et al. (2015), there are many difficulties in achieving adhesions to self-control.

Systematic reviews focused on the study of the maintenance of weight loss, by increasing physical activity or dietary control, reported that initial weight loss was regained after 1 year, or its effects were modest and heterogeneous (Curioni and Lourenço, 2005; Dombrowski et al., 2014). A deeper analysis of behavioral changes in individuals experiencing weight loss or maintenance would help design more successful interventions.

Long-term weight management may involve critical intraindividual changes. It is theorized that self-weighing can induce a change in the behavior of the individual, either in the decision making concerning healthy foods, caloric restriction, or increased physical activity (Dominika et al., 2017; Godino et al., 2019). Although the Guidelines recommend identifying obesity in patients’ diagnoses, there are few treatment options in the Primary Health Care Systems (National Institute for Health and Clinical Excellence, 2006). Self-monitoring has been included in the recommendations from the United States Preventive Services Task Forces as a key tool in the long-term treatment of obesity (Yao, 2012).

The use of behavioral strategies usually develops in two areas, which are the establishment of objectives and self-control (Burke et al., 2011). Regarding self-control, the theory of temporal self-regulation takes into account the momentary influences on health behavior (Hall and Fong, 2007; Dominika et al., 2019). Monitoring the progression of weight loss, not only in face-to-face consultation but also via self-weighing, provides to overweight patients a tool to control the evolution of, and could indirectly assess the adherence to a diet (Hernández-Reyes et al., 2020). The interest in understanding the effectiveness of performing strategies that allow patients to self-control their weight is current (Van Dorsten and Lindley, 2015). Although it has been emphasized as a useful tool (Berk et al., 2018), self-control of body weight is not generally accepted by official institutions (U. S. Department of Health and Human Services and and U. S. Department of Agriculture, 2015).

Evidence suggests that frequent self-weighing is associated with lower body mass index (Gavin et al., 2015; Pacanowski et al., 2015), and is helpful for individuals with obesity to lose weight (Van Wormer et al., 2012). However, the data available in this field are mostly cross-sectional studies and clinical trials that involve women. It is known that, unlike men, women are more predisposed to report dieting and being concerned with their weight (Neumark-Sztainer et al., 1999) and the self-weighing-restraint would be stronger in those (Pacanowski et al., 2019). Therefore, the behavioral effect in the male population should be studied further to elucidate how weight also affects men during weight loss. The extensive review of Shieh et al. (2016), focused on the study of self-weighing in weight management interventions, founded that women were over-represented in the articles.

Although self-weighing has been recognized as a strategy for weight loss and control (Van Wormer et al., 2012; Heather et al., 2013), and is considered component of the cognitive-behavioral approach in weight loss therapy (Elfhag and Rössner, 2005; Berk et al., 2018), it is not well investigated until date.

This study aimed to: (1) determine if the inclusion of the self-weighing, involves or not additional weight loss in men, (2) evaluate the effectiveness in weight loss and body composition of a hypocaloric diet.

## Materials and Methods

### Participants and Setting

Overweight or obese men aged 30–50 years were recruited via advertisements also social media, and included in a complete program based on evidence to lose weight through diet, physical activity, and behavioral changes.

Patients attended an orientation session and completed each self-report evaluation as part of the standard clinical care. This consists of a personal interview to record health status and lifestyle habits, including eating behavior and food preferences, job schedules, and physical activity. The informed consent of each patient was recorded during the first session.

The inclusion criteria were: (i) be at least 18 years old, (ii) BMI between 25 and 50, (iii) own a smart mobile phone with Android or iPhone operating system, and (iv) not have been on a diet to lose weight during the 6 months before the beginning of the study. The exclusion criteria were: (i) be on treatment for diabetes either with oral drugs or insulin injections, due to the variability in the lost or gained weight that the pharmacology for this pathology can cause (Wilding, 2018), (ii) chronic kidney disease, because the macronutrient distribution suggested in the diet, is not recommended for patients with this disease (Bilancio et al., 2019), and (iii) take medications that might affect weight loss.

A total of 70 patients were finally included in the study following the selection criteria. At the end of the study, 54 patients completed the weekly follow-up for 6 months [28 men in the control group (CG) and 26 men in the intervention group (IG)]. The Ethics Committee approved the study of the University of Córdoba (Act n°284, ref.4156) and registered in the Clinical Trial (NCT04032249). Data were collected from February 2018 to December 2018.

The sample size was calculated using Fleiss equation, for a power of 80%, a two-sided significance level of 95% and expecting that 5% of the men who do not receive self-weighed prescription lose weight while this figure will reach 40% in those receiving self-weighed prescription (control or intervention). Although sample resulted in 51 individuals, a size of 70 men (35 for each group), was finally estimated, to mitigate the effect of possible losses during this trial.

### Randomization Group

**FIGURE 1:**
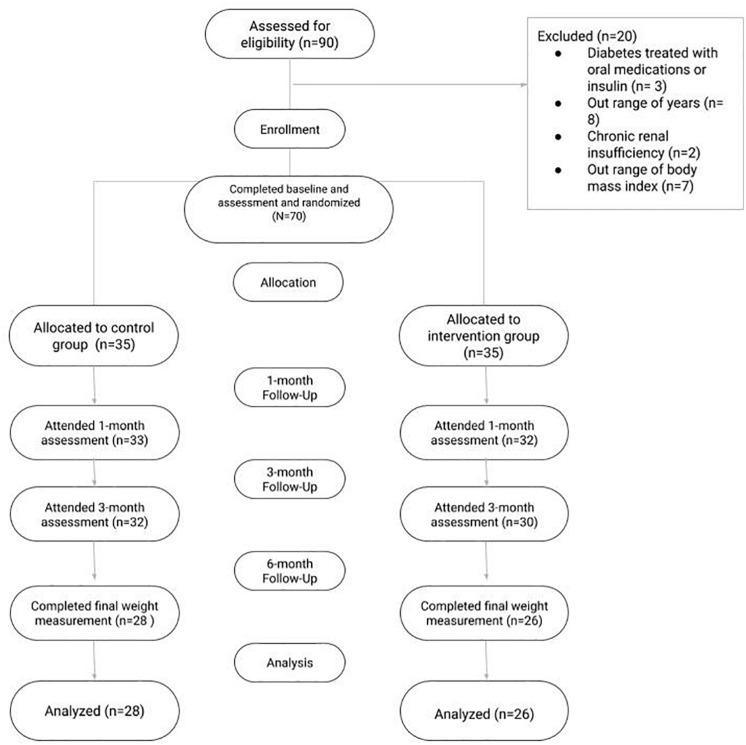
Consort diagram.

Patients were randomly allocated (1:1) to a control or IG using a computerized random-number generator (Figure 1).

The mobile application (app) developed for the study “Nutrición Sur” was installed in both groups (Nutrición Sur, 2017). The screenshot from the app used in the study can be found in the Supplementary Material. The app aims to offer information on the historical record of body composition taken in the clinic, knowledge on healthy habits, and in the case of the IG, access to a specific menu.

A total of 24 face-to-face sessions, lasting 20 min with individual patients, were carried out through 6 months. Each week, patients went to the clinic with an assigned appointment with the nutritionist. The patients of the IG (*n* = 26) had access to a specific menu in the app called “Self-control,” in which they could enter their self-weight, either on their own decision or by request of the dietitian-nutritionist. The IG had educational and individualized motivating content to improve self-management. The CG (*n* = 28) received the same nutritional and educational advice and the establishment of a weight target to reach in the weight loss program, but patients should not be self-weighted, except when they attended the interview once a week.

### Outcomes Measures

#### Self-Weighing

The patients in the IG had to enter their weight in the app when they received push notifications, with a frequency of between one and two times per week. This message was sent if the patient did not previously enter the weight. The details of the methodology regarding the sending of push notifications have been previously published (Hernández-Reyes et al., 2019).

#### Study Variables and Measurements

Body fat, muscle mass, and visceral fat were obtained using multifrequency bioelectrical impedance (BWB-800A, Tanita Corp., United States). This method is currently accepted “gold standard” comprises the four-compartment model previously validated (Schubert et al., 2019). The independent variables were: age (years), height (cm), weight (kg), and BMI. We calculated BMI using the standard equation: weight (kg)/height^2^ (m). The anthropometric measures were registered following the instructions of the reference manual of standardized anthropometry (Callaway et al., 1991).

#### Dietary

Concerning diet, the daily energy requirements were determined by estimating the energy expenditure at rest through the formula proposed by Harris–Benedict. The version of the Harris–Benedict equation for men is as follows, with W equal to weight in kilograms, H the height in centimeters, and A the age in years: 66.47 + (13.75 × W) + (5 × H) - (6.75 × A) (Harris and Benedict, 1919). All the participants were on the same dietary regimen for 24 weeks, with the following distribution of macronutrients: 25–30% proteins, 40–45% carbohydrates, and 30–35% fats. The moderate-fat, restricted-calorie, Mediterranean diet was rich in vegetables and low in red meat, with poultry and fish replacing beef and lamb, with a goal of no more than 35% of calories from fat. The diet is based on the recommendations of Willett and Skerrett (2001). Individualized calorie targets (–500 kcal/day less than estimated expenditure) during the treatment period to achieve a weekly weight loss of 400 g, an amount that is a safe, achievable, and clinically meaningful goal for weekly weight loss (Byrne et al., 2006). No vitamins or other nutritional supplements were prescribed. A proposed menu valid for 7 days was given to the patient and changed weekly for a new protocol, after the review appointment. The energy and nutritional contribution were assessed through the Dietowin^®^ software and the weighing method (Dietowin, 1991–2015).

#### Statistical Analysis

Data are expressed as mean ± standard deviations. To study differences based on categorical variables, the percentages were considered, the analysis is done by means of χ2. An analysis of variance (ANOVA) was conducted to study differences concerning the different variables included in the study, after having applied the Kolmogorov–Smirnoff test in order to analyze whether the data fitted normal distribution, all distributions were sufficiently normal. For all statistical analyses, an alpha error probability of less than 5% was accepted (*p* < 0.05), and the confidence interval was calculated with 95% confidence. For the statistical analysis, IBM SPSS Statistics software version 22.0 was used.

## Results

### Anthropometric Measurement at the Beginning of the Intervention

The characteristics of the participants at the beginning of the intervention are shown in Table 1. The 54 men had a mean age of 40.1 ± 11.1 years. Concerning body composition, the average percentage of body fat was 29.7 ± 4.8%, muscle mass, expressed in kilograms, 69 ± 6.8 and visceral fat score had an average value of 13.5 ± 4.6. None of the analyzed variables showed significant differences (*p* > 0.05) at the beginning of the intervention between the study groups.

**TABLE 1:** Anthropometric measurement at the beginning of the intervention.

Variable	Total	Intervention (*n* = 26)	Control (*n* = 28)	*p*
	Mean (± SD)	Mean (± SD)	Mean (± SD)	
Age (years)	40.1 (11.1)	39 (11.1)	41 (11.1)	0.490
Height (m)	1.77 (0.07)	1.76 (0.05)	1.77 (0.08)	0.732
Weight (kg)	102.9 (13.9)	100.8 (14.5)	104.9 (13.2)	0.285
BMI (kg/m^2^)	32.9 (3.9)	32.4 (4.5)	33.4 (3.5)	0.342
Body fat (%)	29.7 (4.8)	28.8 (5)	30.5 (4.4)	0.185
Muscle mass (kg)	69 (6.8)	68.7 (6.7)	69.3 (7)	0.734
Visceral fat	13.5 (4.6)	12.9 (4.6)	14.1 (4.6)	0.334

*BMI, body mass index.*

### Evolution of Body Composition Throughout the Intervention

Table 2 shows the evolution of body composition of all men who completed the study and were included in the data analysis. Regardless of the group to which they belonged, the men showed significant weight loss throughout the 24-week follow-up (*p* < 0.001). The loss of body fat showed similar behavior, reducing significantly from the beginning to the end of the intervention (*p* < 0.001). No significant variations were observed in muscle mass between 24 weeks of follow-up (*p* = 0.222). Finally, the reduction of visceral fat was also significant throughout the 24 weeks of the intervention.

**TABLE 2:** Evolution in the 24-week follow-up with respect to baseline (*n* = 54).

Variables	Week 4	Week 8	Week 12	Week 16	Week 18	Week 24	*p*
	mean (±SD)	mean (±SD)	mean (±SD)	mean (±SD)	mean (±SD)	mean (±SD)	
Weight (kg)	−3.5 (2.1)	−4.9 (2.6)	−6 (3.5)	−6.5 (3.6)	−7 (3.9)	−7.1 (4.1)	<0.001
Body fat (%)	−3.6 (4.7)	−6.4 (8)	−7.5 (8)	−9.2 (8.6)	−10.9 (9)	−11.8 (10.7)	<0.001
Muscle mass (kg)	−2.6 (5.3)	−3 (5.5)	−3.4 (6.1)	−3.7 (5.4)	−3.5 (5.6)	−3.5 (5.5)	0.222
Visceral fat	−6.4 (7.7)	−10.5 (9.4)	−12.7 (11.8)	−14.8 (12.4)	−15.9 (12.9)	−17.4 (13.9)	<0.001

### Evolution of Body Composition According to the Assigned Group

When the group assigned after randomization was introduced in the analysis, its influence was significant in weight loss (*F*1.52 = 19.465, ± 2 = 0.272, *p* < 0.001). A decrease in body fat percentage (*F*1.52 = 8,306, ± 2 = 0.132, *p* < 0.01) and visceral fat (*F*1.52 = 14.285, ± 2 = 0.216, *p* < 0.001) were also found in the IG. In the case of the variation in muscle mass, the effect was not significant at the end of the study (*F*1.52 = 2.574, ± 2 = 0.047, *p* = 0.115). In Tables 3A,B, comparisons can be observed in each measurement based on the membership group.

**TABLE 3A:** Group comparison in the evolution in body composition at weeks 4, 8, and 12 depending on the reception of notifications for self-weighing or not.

Variables	Week 4		Week 8		Week 12	
	Intervention	Control	Intervention	Control	Intervention	Control
	Mean (±SD)	Mean (±SD)	Mean (±SD)	Mean (±SD)	Mean (±SD)	Mean (±SD)
Weight (kg)	−4.3(2.1)**	−2.9(1.9)	−6.1(2.5)**	−3.8(2.3)	−7.7(3.4)***	−4.3(2.9)
Body fat (%)	−4.4(5)	−2.9(4.3)	−8(6.7)	−4.9(6.8)	−9.6(8.2)	−5.6(7.4)
Muscle mass (kg)	−3.5(6.8)	−1.8(3.3)	−4(6.7)	−2.1(3.9)	−5.1(6.9)*	−1.8(4.8)
Visceral fat	−9.5(8.3)**	−3.6(5.9)	−13.9(9.9)**	−7.2(7.6)	−17.7(12.1)**	−8.1(9.5)

*p < 0.05, **p < 0.01, ***p < 0.001. The comparisons are between pairs of measurements of the same week (intervention vs. control).

**TABLE 3B:** Group comparison in the evolution in body composition at weeks 16, 20, and 24 depending on the reception of notifications for self-weighing or not.

Variables	Week 16		Week 20		Week 24	
	Intervention	Control	Intervention	Control	Intervention	Control
	Mean (±SD)	Mean (±SD)	Mean (±SD)	Mean (±SD)	Mean (±SD)	Mean (±SD)
Weight (kg)	−8.4(3.1)***	−4.7(3.1)	−9(3.4)***	−5.1(3.5)	−9.2(3.5)***	−5.1(3.5)
Body fat (%)	−12.7(7.5)**	−5.9(8.2)	−14.8(7.1)**	−7.3(9.2)	−15.3(10.4)*	−8.5(10.1)
Muscle mass (kg)	−4.8(6.9)	−2.7(3.5)	−4.7(6.9)	−2.4(3.8)	−4.8(6.7)	−2.2(3.8)
Visceral fat	−19.9(12.7)**	−10(10.2)	−21.6(12.7)**	−10.7(11.1)	−24.1(12.9)***	−11.1(17.8)

*p < 0.05, **p < 0.01, ***p < 0.001. The comparisons are between pairs of measurements of the same week (intervention vs. control).

**FIGURE 2:**
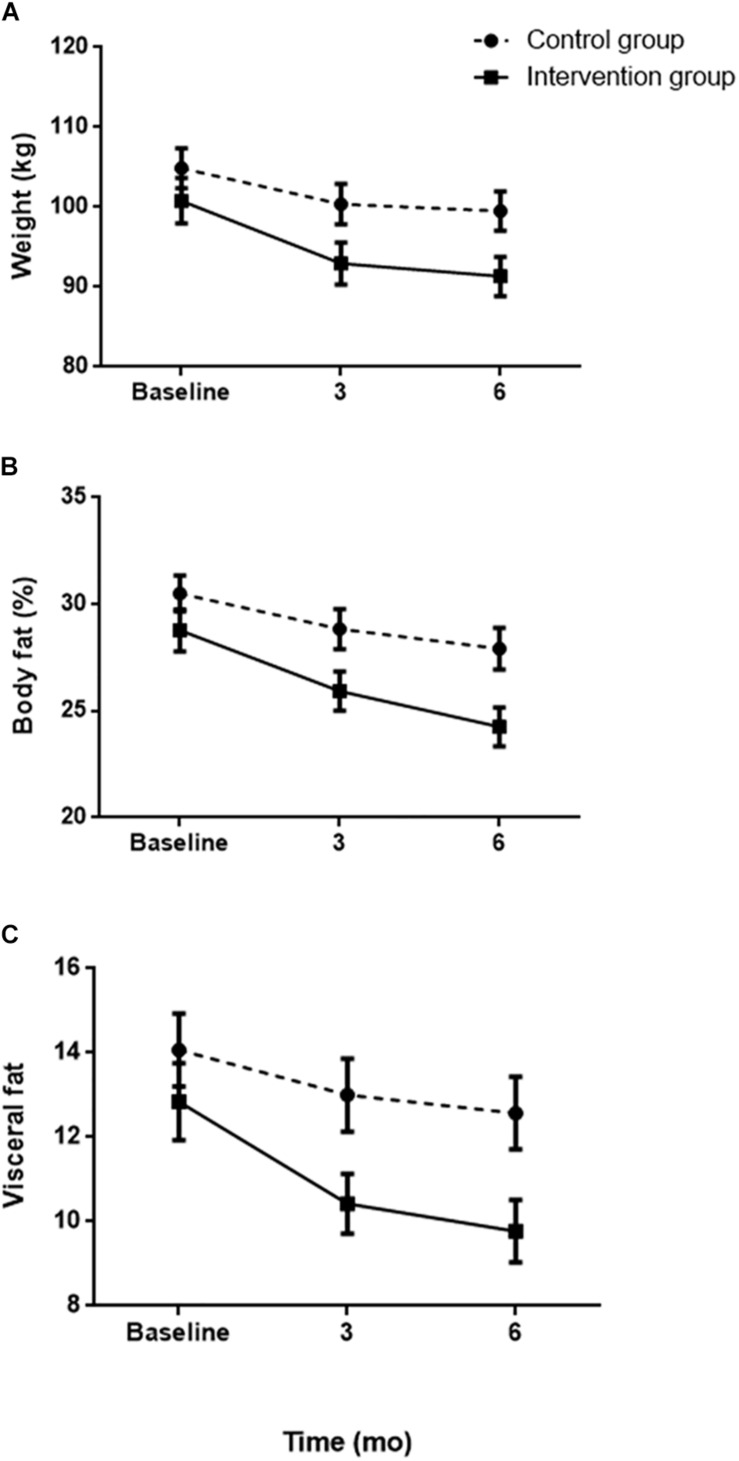
Evolution of weight **(A)**, body fat **(B)**, and visceral fat **(C)** according to the assigned group.

Throughout the entire intervention, it will be seen that the weight loss was greater in the group that performed self-weighing. The difference between both groups increased from week 12, reaching week 24, a mean difference (MD) of 4.1 (*F*1.52 = 18.885, *p* < 0.001) (Figure 2A). The reduction in body fat did not select the same trend as that observed in body weight. During the first 12 weeks, although those who performed self-weighing reduced their body fat percentage more than control, the changes produced between groups were not significant (*p* = 0.062). However, as of week 12, it was detected that the fat loss began to be significantly greater in the group that received the messages at week 16 (MD = 6.8, *F*1.52 = 10.095, *p* < 0.01), the week 20 (MD = 7.5, *F*1.52 = 11.277, *p* < 0.01), and at week 24 (MD = 6.8, *F*1.52 = 6.015, *p* < 0.05) (Figure 2B).

Although no significant differences were found in the results of muscle mass, patients subjected to intervention lost an additional 2.6 kg (±1.45) of muscle at the end of the study. Finally, visceral fat experienced significant reductions in those who performed self-weighing from week 4 (MD = 5.9, *F*1.52 = 9.133, *p* < 0.01) to week 24 (MD = 12.9, *F*1.52 = 14.720, *p* < 0.001) (Figure 2C).

## Discussion

We examined the effect on weight change of including self-weighing in patients who attended face-to-face consultation, under the same hypocaloric dietary prescription, and checked once a week. Patients in the IG were controlled both by the nutritionist and by themselves. Our results show how weighing at home and having knowledge of their weight can be associated with a lower BMI and more significant weight loss.

In our study, both CG and IG, the hypocaloric diet was associated with an improvement in body composition, reflected in a decrease in total body weight, total body fat, and visceral fat score. At 12 weeks, both groups obtain reductions in weight between 5 and 10%, a fact that should be recognized as relevant from a clinical health point of view (Hooper, 2007), and that matches with the general recommendations of an objective to be achieved in weight loss programs (Phillips, 2014).

As recently published (Hernández-Reyes et al., 2019, 2020), a hypocaloric diet is a useful therapy to lose weight in the short term even in sedentary people. However, in the long term, other strategies must be combined with caloric restriction. In our results, when the progression of the body composition was studied, the same tendency was observed in the loss of weight and visceral fat in men, with a reduction after 3 months of treatment but not from 3 to 6 months. These results confirm that the effectiveness of the hypocaloric diet in weight loss programs is limited by time, requiring adjuvant therapies for long-term treatment.

As previous reviews and studies have already reported, adding self-weighing to the dietary prescription implies increased weight loss (Chambers and Swanson, 2012; Zheng et al., 2015). However, the authors recognize as methodological weakness the way of how patients evaluated their weight. Most studies used self-reported methods and asked participants about the frequency of self-weight (e.g., daily, weekly), which might not accurately reflect their actual weighing behavior. In this sense, our study improves previous methods and shows the effect of the self-weighing as an objective tool. In the reviews and studies mentioned, the sample was predominantly composed of middle-aged white women. This limits the generalization of findings and understanding of acceptability, compliance, and the effect of self-weight among men.

According to an extensive review by Pacanowski et al. (2015), patients who have psychological disorders tend to over-control their weight and also tend to fail in therapy. Therefore, the benefits of self-weighing may be closely related to weight change, rather than self-weighing *per se*. In this way, our study shows that, even when people enroll in a weight loss program, the introduction of self-control of weight greatly improves the results in bodyweight reduction and better restores body composition when compared to those who do not use this tool. These results strengthen that self-weighing has positive effects, but also needs to be controlled by the clinician.

Although we have seen that self-weight can be an additional tool to help weight loss, it should be borne that people with obesity have a greater tendency to suffer from eating disorders (Udo and Grilo, 2018). Weight self-control during the weight-loss period has been considered in previous research as positive concerning the increase in personal satisfaction (Welsh et al., 2009). This aspect could explain why patients subjected to the intervention had better results in weight reduction during the 6 months lasted our study. In the work of Steinberg et al. (2014), in which the feelings that the self-control of the weight was evaluated, the participants did not report negative experiences, frustration, or feel self-conscious.

The instructions for self-weighing emphasized on the importance of regular weighing. The results suggest that assigning role of the weight control to the patient implies an additional engagement in weight loss programs; for example, by a greater dietary self-control, increasing the degree of adherence (Pacanowski et al., 2015), and/or being more physically active (Harkin et al., 2016). Although the patients did not have any additional prescription of physical activity, it is possible that controlling the total body weight regularly, it reinforces a behavioral change in this sense, as previous studies have shown (Wang et al., 2012).

According to our results, while the hypocaloric diet is effective in both groups during the first 12 weeks. No significant differences were found in this period in the weight loss between the two groups. At the end of the program, the IG patients lost 4.5 additional kilograms of total body weight compared to the CG. Our findings coincide with previous experimental works in which the eating behavior, for example, the conscious restriction of food intake as a means of weight control; disinhibition, which is the tendency to overeat in response to different stimuli, in weight loss was studied (Batra et al., 2013; Steinberg et al., 2013). Again, the importance of including behavioral elements in the treatment of weight loss is emphasized, instead of focusing exclusively on dietary intervention. Checking the progress of lost weight can be a reason for introducing changes in healthy habits in the lifestyle of overweight patients to get better results (Claire et al., 2015). Moreover, our outcomes prove that, concerning BMI, weekly self-weighing held approximately 1 and 2 BMI unit advantages, results that coincide with the review by Van Wormer et al. (2008).

Our study elucidates how important is to introduce self-weighing in weight loss therapy. Our results show that body fat was not significantly reduced until patients reach week 16, unlike visceral fat that began to be significant on week 4. In this sense, visceral fat has been identified as a marker associated with chronic back pain (Brooks et al., 2016), insulin resistance, and metabolic syndrome (De Lucia et al., 2015), which suggests that the advantages of self-weighing could play an important role in public health by decreasing abdominal fat.

### Limitations and Strengths

The strengths of the study include similar sample size, the repeated measures of self-weighing behavior in common across the studies, and weight were objectively measured with traditional and face-to-face treatment. Several factors related to the evaluation limited the study design. The frequency of self-weighing is not defined, and patients must have weight control with at least a frequency of once a week, but without control over whether patients are weighed more frequently. We also impaired to study the age-dependent effect. In this sense, our study population was included in a narrow range of age that did not allow to assess this point.

## Conclusion

As the results of this study suggest, self-weight can induce further significant weight loss and considerably improve body composition. In the light of our findings, the effectiveness of regular weight monitoring, in the form of self-weighing, must be determined rigorously. Clinical and public health recommendations for self-weight should be considered.

## Data Availability Statement

The datasets generated for this study are available on request to the corresponding author.

## Ethics Statement

The studies involving human participants were reviewed and approved by the Ethics Committee of the University of Córdoba (Act n°284, ref.4156). The patients/participants provided their written informed consent to participate in this study.

## Author Contributions

AH-R designed the study and supervised the data collection. RM-L performed the statistical analysis. FC-M, ÁV, and RM-R contributed to data analysis and interpretation. All authors wrote the manuscript and approved the final version.

## Conflict of Interest

The authors declare that the research was conducted in the absence of any commercial or financial relationships that could be construed as a potential conflict of interest.
