# Supplementary figures and images for: Effects of Self-Weighing During Weight Loss Treatment: A 6-Month Randomized Controlled Trial
Source: Front Psychol. 2020 Mar 10;11:397. doi: 10.3389/fpsyg.2020.00397 (PMC7077514; doi:10.3389/fpsyg.2020.00397)

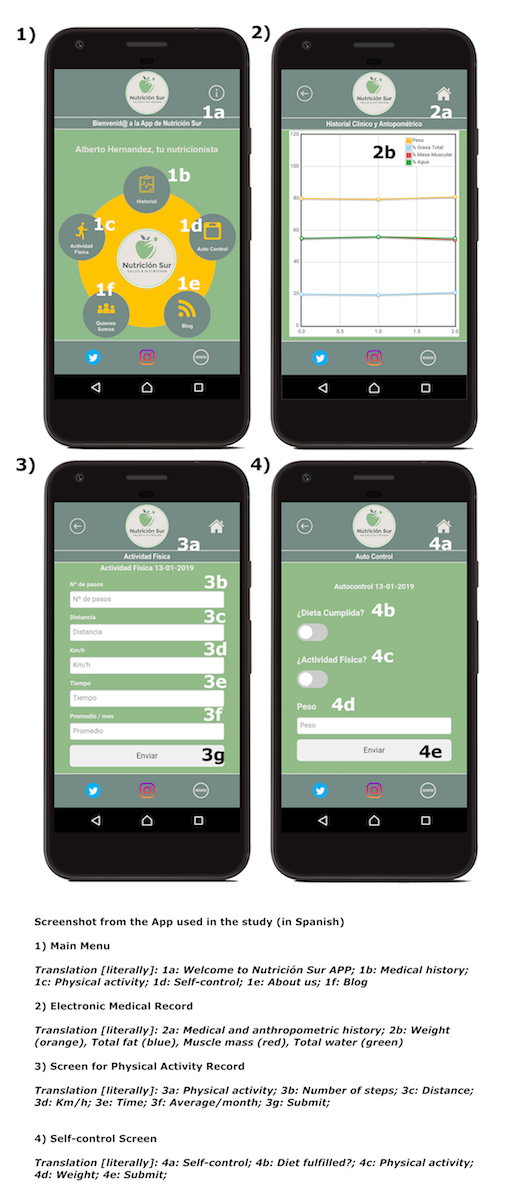

Supplement: Supplementary file 1 [file Image_1.PNG]
